# Antimicrobial resistance and virulence of subgingival staphylococci isolated from periodontal health and diseases

**DOI:** 10.1038/s41598-023-38599-4

**Published:** 2023-07-18

**Authors:** Ana Paula Vieira Colombo, Renata Martins do Souto, Lélia Lima Araújo, Laís Christina Pontes Espíndola, Fátima Aparecida R. R. Hartenbach, Clarissa Bichara Magalhães, Gabrielle da Silva Oliveira Alves, Talita Gomes Baêta Lourenço, Carina Maciel da Silva-Boghossian

**Affiliations:** 1 Institute of Microbiology, Department of Medical Microbiology, Federal University of Rio de Janeiro, Rio de Janeiro, Brazil; 2 Department of Clinics, School of Dentistry, Federal University of Rio de Janeiro, Rio de Janeiro, Brazil

**Keywords:** Microbiology, Diseases

## Abstract

The dysbiotic biofilm of periodontitis may function as a reservoir for opportunistic human pathogens of clinical relevance. This study explored the virulence and antimicrobial susceptibility of staphylococci isolated from the subgingival biofilm of individuals with different periodontal conditions. Subgingival biofilm was obtained from 142 individuals with periodontal health, 101 with gingivitis and 302 with periodontitis, and cultivated on selective media. Isolated strains were identified by mass spectrometry. Antimicrobial susceptibility was determined by disk diffusion. The *mecA* and virulence genes were surveyed by PCR. Differences among groups regarding species, virulence and antimicrobial resistance were examined by Chi-square, Kruskal–Wallis or Mann–Whitney tests. The overall prevalence of subgingival staphylococci was 46%, especially in severe periodontitis (> 60%; p < 0.01). *S. epidermidis* (59%) and *S. aureus* (22%) were the predominant species across groups. *S. condimenti, S. hominis, S. simulans* and *S. xylosus* were identified only in periodontitis. High rates of resistance/reduced sensitivity were found for penicillin (60%), amoxicillin (55%) and azithromycin (37%), but multidrug resistance was observed in 12% of the isolates. Over 70% of the *mecA* + strains in periodontitis were isolated from severe disease. Higher detection rates of *fnB* + isolates were observed in periodontitis compared to health and gingivitis, whereas *luxF/luxS-*pvl + strains were associated with sites with deep pockets and attachment loss (p < 0.05). Penicillin-resistant staphylococci is highly prevalent in the subgingival biofilm regardless of the periodontal status. Strains carrying virulence genes related to tissue adhesion/invasion, inflammation and cytotoxicity support the pathogenic potential of these opportunists in the periodontal microenvironment.

## Introduction

Species of the genus *Staphylococcus* are commensal inhabitants of the skin and mucosal microbiota of humans and animals^1^. In certain clinical conditions, such as long-term hospitalization, immunosuppression, ageing and comorbidities, *Staphylococcus aureus* and the coagulase negative staphylococci (CoNS) *Staphylococcus epidermidis* and *Staphylococcus haemolyticus* are among the most prominent opportunistic human pathogens responsible for a variety of community-associated and nosocomial infections^2^, particularly biofilm-related infections of indwelling medical devices^3^. Currently, staphylococci have become a global health threat due to their relevant role in the dissemination of multidrug resistance (MDR)^4,5^. In addition, staphylococcal pathogenicity is determined by a broad spectrum of virulence factors^2,6^.

The recognition of *Staphylococcus* spp. as a true colonizer of the oral microbiota, as opposed to a mere contaminant is still controversial^7–10^. These species have been detected in the dental biofilm at different frequency rates^10–12^, particularly in the dysbiotic biofilm associated with periodontal diseases (PDs) and peri-implantitis^13–22^. Evidence also suggests that staphylococci are frequently isolated from angular cheilitis, osteomyelitis of the jaw, parotitis, endodontic infections and mucositis^23–25^. Therefore, the microbiota associated with oral diseases may function as a reservoir for several pathogens of clinical medical importance, including multidrug resistant staphylococci^7–9,11^.

Periodontal disease is a chronic inflammatory disease that results from the overgrowth and establishment of a dysbiotic polymicrobial biofilm. Destructive forms of disease lead to loss of periodontal attachment, alveolar bone destruction, and eventual tooth loss^26,27^. Moreover, the onset, progression and severity of periodontal diseases are modulated by various genetic, environmental and behavioral host-related factors^28^. The dysbiotic subgingival biofilm in periodontal diseases may allow the colonization and favor the overgrowth of a large variety of pathogenic microorganisms not usually considered members of the oral microbiota^18,29–31^. The potential role that these opportunistic pathogens may have in the dysbiosis of the periodontal biofilm and/or in the pathogenesis of these diseases is unknown^8,13^, but growing within such a rich biofilm may be advantageous^32^. They can interact with more adapted oral species, becoming less susceptible to antimicrobials and the immune system, which increases the risk of therapeutic failure^33,34^. In the current context of the link between periodontal and systemic diseases, periodontal pockets harboring high counts of opportunistic pathogens could be seen as a potential infectious focus for hematogenous dissemination and development of infections in other parts of the human organism, particularly in hospitalized, elderly and immunocompromised individuals^9,11,35^. However, the oral cavity is still rarely considered a potential source of such pathogens^8,9^.

In this study, the prevalence and antimicrobial susceptibility profile, as well as the virulence genes of staphylococci species isolated from the subgingival biofilm of patients with periodontal health and diseases were investigated. Moreover, these parameters were correlated with the clinical and demographic features of the sample population.

## Materials and methods

### Study population

This cross-sectional study was conducted in full accordance with the Helsinki Declaration and approved by the Human Research Ethics Committee of the Hospital at Universidade Federal do Rio de Janeiro (UFRJ), Brazil (#3.941.981). Adult individuals presenting at least 16 teeth, who attended the Division of Graduate Periodontics of the School of Dentistry at UFRJ between March/2008 and December/2019 were recruited. To participate, individuals were informed about the risks and benefits of the study and signed an informed consent form. Exclusion criteria were the existence of chronic inflammatory systemic diseases, use of topical or systemic antimicrobials in the last 6 months, use of anti-inflammatory drugs in the last 3 months, periodontal therapy in the last 6 months, need for antibiotic prophylaxis, orthodontic treatment, smoking or smoking cessation < 10 years, pregnancy or nursing. Eligible individuals were clinically examined and diagnosed with periodontal health, gingivitis or periodontitis, as previously described^36^. Briefly, periodontitis was defined as ≥ 10% of teeth with probing pocket depth (PPD) and/or clinical attachment level (CAL) ≥ 5 mm or ≥ 15% of teeth with PPD and/or CAL ≥ 4 mm and bleeding on probing (BOP). Gingivitis was defined as ≥ 10% of bleeding sites (BOP or marginal gingival bleeding—Gb) with PPD ≤ 3 mm, on an intact or reduced/stable periodontium in a patient without a history of periodontitis. Periodontal health was defined as < 10% of sites with BOP, no PPD or CAL > 3 mm, although PPD or CAL = 4 mm in up to 5% of the sites without BOP. Patients within the periodontitis group were further classified by disease severity according to the current periodontal disease classification guidelines^26^.

### Sample size calculation

Considering the prevalence of subgingival staphylococci as the primary outcome, unpublished data of our group indicated differences of 14.8 ± 2.9% and 13.7 ± 3.7% in the mean frequency ± standard error of these microorganisms between patients with health and periodontitis, and health and periodontal diseases (gingivitis + periodontitis), respectively. The sample size was calculated based on an estimated alpha error of 5%, 90% of power, a 2:1 case/control ratio and one tail to detect the lowest difference (14%) between health and periodontal diseases. A total of 199 individuals in the disease group and 99 in the health group were estimated (G*Power version 3.1.9.7, Kiel University, Germany).

### Clinical examination

Clinical measurements were performed by trained and calibrated periodontists, with intra-class correlation coefficients > 0.85 for PPD and CAL. Periodontal parameters were measured using a manual periodontal probe (UNC-15, Hu-Friedy, Chicago, IL, USA) at 6 sites/tooth of all teeth, except the third molars. PPD and CAL were recorded to the nearest millimeter, whereas the percent of sites with the presence or absence of visible supragingival biofilm (Sb), dental calculus (Ca), gingival bleeding (Gb), bleeding on probing (BOP) and suppuration (Sup) were computed per patient^36^. Demographic data and medical/dental health history were obtained by questionnaires. Individuals with periodontal diseases and other dental needs were referred for treatment in the Dental Clinic of the School of Dentistry of UFRJ.

### Isolation and identification of staphylococci from subgingival biofilm

After removal of supragingival plaque, subgingival biofilm samples were taken with sterile Gracey curettes (Hu-Friedy) from eight selected periodontal sites of each patient, including posterior and anterior teeth from all quadrants, as previously described^37^. In healthy individuals, samples were taken from healthy sites; in patients with gingivitis, from sites with bleeding, but no periodontal pocket; and in periodontitis patients, from sites with the deepest periodontal pockets. The 8 samples/patient were pooled into a vial containing cryogenic Mycoplasma broth with 5% dimethyl sulfoxide (Sigma-Aldrich Brasil Ltda, São Paulo, Brazil) for immediate processing. Samples were vigorously mixed and inoculated into Tryptic soy broth with 6.5% of NaCl and Brain heart infusion broth (Bacto™ Becton, Dickinson and Company, Sparks, MD, USA), at 37 °C for 48–72 h. Cultures with microbial growth were subsequently plated on Mannitol Salt agar media (BBL™ Becton, Dickinson and Company) and incubated at 37 °C, for 48 h. Colonies grown on plates were isolated, checked for purity, cell morphology, Gram staining and catalase production, and transferred to pure culture to be identified by mass spectrometry (MALDI-ToF; MicroflexLT; Bruker Daltonics, Billerica, MA, USA). Data acquisition and evaluation for species identification were obtained using the software Biotyper versão 3.1 (Bruker Daltonics). The strain *Escherichia coli* DH5α was used as a standard strain for calibration. The spectra of samples were compared to the standard spectra in the MS database, resulting in scores between 0 and 3.0. The higher the score, the greater the identification reliability. The MALDI-TOF MS technology has been widely used as a reliable, accurate, rapid and inexpensive method for bacterial identification^38^. Accuracy and reproducibility of this technology for detection and identification of clinical isolates of staphylococci at the species level have been shown to be very high (> 93%)^39^. For this study, only strains with scores ≥ 2.0 were considered as species. Few strains (7) with scores ≥ 1.7 were identified at the genus level, even after 2–3 analyses. Isolates were cultivated for DNA extraction and for storage in cryogenic broth at – 80 °C.

### Antimicrobial susceptibility analysis

Antimicrobial susceptibility was carried out by disk diffusion according to the Clinical and Laboratory Standards Institute recommendations^40^. Antimicrobials tested included penicillin G (10 U), amoxicillin (10 µg), amoxicillin-clavulanic acid (20/10 µg), cefoxitin (30 µg), azithromycin (15 µg), ciprofloxacin (5 µg), moxifloxacin (5 µg), clindamycin (2 µg), doxycycline (30 µg), minocycline (30 µg), chloramphenicol (30 µg), cotrimoxazole (sulfamethoxazole-trimethoprim 1.25/23.5 µg), gentamicin (10 µg), linezolid (30 µg) and rifampin (5 µg). Isolated strains were cultivated on Tryptic soy agar (Becton, Dickinson and Company) for 18–24 h at 37 °C. Bacterial suspensions were prepared in 0.85% saline solution to reach a 0.5 McFarland turbidity standard and inoculated onto Müeller-Hinton agar (BBL™ Becton, Dickinson and Company) to achieve confluent growth. Antimicrobial-containing discs (Cecon, São Paulo, Brazil) were deposited onto inoculated plates and the inhibition zones were measured after 16–18 h of incubation at 35 °C. The strain *S. aureus* ATCC^®^ 25923 was tested as a control. MDR was defined as non-susceptibility to at least one agent in three or more antimicrobial categories^41^.

### Detection of methicillin-resistance (*mecA*) gene

Genomic DNA was obtained and purified from pure cultures of each isolated strain, and a multiplex PCR protocol^42^ was carried out for the detection of the *mecA* gene and confirmation of the identity of *S. aureus* and CoNS. Amplification was performed in a 100 µL reaction containing ~ 100 ng of template DNA, 10 µL of 10xPCR buffer (200 mM Tris–HCl, pH 8.0; 500 mM KCl), 0.2 mM dNTPs, 1.5 mM MgCl_2_, 2.5 U of *Taq* polimerase (Promega Biotecnologia do Brasil LTDA, São Paulo, Brazil), and 10 pmol of each primer (Appendix Table S1). The protocol program included an initial denaturation step at 94 °C for 3 min, followed by 36 cycles of a denaturation step at 94 °C for 1 min, a primer annealing step at 55 °C for 1 min, an extension step at 72 °C for 1 min, and a final step of 72 °C for 10 min (Axygen^®^ Maxygene Gradient thermal cycler, Union City, CA, USA). Purified DNA from *S. aureus* ATCC® 25,923 and *S. epidermidis* ATCC^®^ 14579 were included as controls. Amplicons were visualized on a 1.5% agarose gel on an UV transilluminator (MiniBis Pro, Bio-Imaging System, Neve Yamin, Israel).

### *Staphylococci* virulence factor genes

Genes related to virulence factors were investigated by PCR protocols described in previous studies^43–45^. The target genes included *clfA* (clumping factor A), *ebpS* (elastin-binding protein), *cna* (collagen-binding protein), *fnbA* and *fnbB* (fibronectin-binding proteins A and B), *bbp* (bone sialoprotein-binding protein), *luxF/luxS-pvl* (Panton–Valentine leukocidin), and *groEL* (chaperonin of the heat shock protein family). Amplifications were carried out in a thermocycler (Axygen^®^), in a 25 µL reaction containing ~ 100 ng of template DNA, PCR buffer (200 mM Tris–HCl, pH 8.0; 500 mM KCl), 0.2 mM dNTPs, 2 mM MgCl2, 1 U of *Taq* polymerase (Promega Biotecnologia do Brasil LTDA), and 10 pmol (*fnA, fnB, ebpS*) or 20 pmol (*can, bbp, pvl*) of specific primers (Appendix Table S1). The thermal cycling conditions included an initial denaturation step (5 min at 94 °C) followed by 30 cycles of denaturation for 1 min at 94 °C, annealing for 1 min at 55 °C (50 °C for *fnbA*), extension for 1 min at 72 °C; and a final 10-min incubation step at 72 °C. For the *luxF/luxS-pvl* genes, the program included 30 cycles of 30 s at 94 °C, 30 s at 55 °C and 30 s at 72 °C. Amplification of the *groEL* gene was carried out in a 100 μL mix reaction containing 100 ng of template DNA, 0.5 μg of each primer, 0.25 mM dNTPs, 1.5 mM MgCl_2_, PCR buffer and 2 U of *Taq* polymerase (Promega). The thermal cycler program was 3 min of denaturation at 95 °C, followed by 40 cycles of 1 min denaturation at 94 °C, 2 min annealing at 37 °C, 1 min elongation at 72 °C, and a final cycle of 10 min at 72 °C. Amplicons were visualized on a 1% agarose gel on a UV transilluminator (MiniBis Pro, Bio-Imaging System, Neve Yamin, Israel).

### Statistical analysis

A large database containing clinical, demographic and microbiological parameters was validated and error proofed by a senior investigator (APVC). Clinical data were computed for each individual, and then across groups. The frequency of detection of staphylococci species, virulence factors, *mecA* gene and antimicrobial resistance were calculated for each patient and within groups. Differences among clinical conditions for the parameters evaluated were sought by Chi-square, Fisher’s exact, Kruskal–Wallis and Mann–Whitney tests. The level of significance was set at 5%. All analyses were performed using statistical software (IBM SPSS^®^ Statistics 21.0, SP, Brazil).

## Results

### Features of the study population

Demographic and clinical data of 545 eligible patients recruited for the study are presented in Table 1. Approximately half of the individuals (42.75%) were culture-positive for staphylococci in their subgingival biofilm samples. In general, diseased patients were older, former/current smokers, of low socio-economic status and of African heritage (Chi-square and Mann–Whitney tests, p < 0.05). Both staphylococci-positive and -negative patients across the clinical groups were similar regarding the parameters evaluated, except for PPD, CAL and Sup, which had higher values in periodontitis patients harboring subgingival staphylococci compared to negative periodontitis individuals (Mann–Whitney test, p < 0.05).

**Table 1:** Demographic and clinical periodontal data of individuals of the study population who were positive or negative for staphylococci.

Parameters	Staphylococci-positive (n = 233)			Staphylococci-negative (n = 312)		
	Periodontal Health	Gingivitis	Periodontitis	Periodontal Health	Gingivitis	Periodontitis
N	44	38	151	98	63	151
Mean (Sd) age in years	29.9 (12.4)^b^	30.3 (12.6)^a^	42.7 (13.2)^a,b^	27.4 (9.9)^b^	28.9 (11.9)^a^	44.5 (12.3)^a,b^
Gender						
% males	31.8	32.5	42.5	27.6	27.0	35.8
% females	68.2	67.5	57.5	72.4	73.0	64.2
Race/color						
% White	71.1^d^	52.5	41.0^d^	70.1^d,e^	43.0^e^	45.5^d^
% African Americans	13.2	12.5	19.9	7.2	19.0	21.4
% Mestizos/Mulattos/Others	15.8	35.0	39.1	22.7	38.0	33.1
% Household monthly income						
Up to 1 minimum wage	10.8	27.5	25.8	5.3	19.7	32.4
Up to 2 minimum wages	5.4	20.0	41.7	12.8	27.9	34.5
> 2 minimum wages	83.8^d,e^	52.5^e,f^	32.4^df^	90.9^d,e^	73.3^e,f^	44.0^d,f^
% Education						
Middle school	2.6	5.1	18.6	1.0	4.8	18.8
High school	13.2	43.6	40.0	10.3	54.8	44.9
Higher education	84.3^d,e^	51.3^e^	41.4^d^	81.9^d,e^	52.5^e,f^	33.5^d,f^
% Smoking						
Never smokers	92.3	90.0	86.8	95.0^d^	90.5^f^	78.0^d,f^
Smokers/former smokers	7.7	10.0	13.2	5.0	9.5	22.0
Mean (Sd) of periodontal parameters						
N of missing teeth	1.4 (2.8)^b^	2.1 (3.5)^a^	3.9 (3.7)^a,b^	0.7 (1.5)^b,c^	1.6 (2.7)^a,c^	3.9 (4.1)^a,b^
PPD (mm)^g^	1.8 (0.3)^b,c^	2.0 (0.3)^a,c^	3.2 (1.1)^a,b^	1.8 (0.4)^b,c^	2.0 (0.2)^a,c^	2.8 (0.8)^a,b^
CAL (mm)^g^	1.8 (0.5)^b^	1.9 (0.4)^a^	3.6 (1.3)^a,b^	1.8 (0.4)^b,c^	2.0 (0.3)^a,c^	3.2 (1.3)^a,b^
% sites with						
PPD > 5 mm^g^	0^b^	0^a^	20.1 (21.5)^a,b^	0^b^	0^a^	11.4 (15.3)^a,b^
CAL > 5 mm^g^	0.3 (1.2)^b^	0.05 (0.3)^a^	16.1 (19.3)^a,b^	0.1 (0.6)^b^	0.01 (0.09)^a^	10.8 (17.6)^a,b^
BOP	4.1 (3.5)^b,c^	20.9 (14.1)^a,c^	43.2 (21.3)^a,b^	3.1 (3.1)^b,c^	21.0 (14.8)^a,c^	38.5 (24.5)^a,b^
Sb	13.2 (13.9)^b,c^	35.2 (17.9)^a,c^	51.8 (21.8)^a,b^	9.8 (11.2)^b,c^	35.7 (15.7)^a,c^	49.2 (23.4)^a,b^
Gb	3.1 (4.2)^b,c^	19.2 (11.6)^c^	24.2 (18.2)^b^	2.3 (3.2)^b,c^	19.9 (9.2)^c^	25.4 (18.9)^b^
Ca	4.6 (5.9)^b,c^	12.6 (18.2)^a,c^	24.7 (17.1)^a,b^	4.3 (7.2)^b,c^	7.2 (6.0)^a,c^	24.7 (19.5)^a,b^
Sup^g^	0^b^	0.02 (0.1)^a^	0.60 (2.4)^a,b^	0^b,c^	0.03 (0.1)^c^	0.42 (1.8)^b^

*PPD* probing pocket depth, *CAL* clinical attachment level, *BOP* bleeding on probing, *Sb* supragingival biofilm, *Gb* gingival bleeding, *Ca* calculus, *Sup* suppuration. Significant differences between periodontitis and gingivigis^a^, periodontitis and health^b^, health and gingivigis^c^ (Mann–Whitney test, p < 0.05). Significant differences between between periodontitis and gingivigis^e^, periodontitis and health^d^, health and gingivigis^f^ (Chi-square test, p < 0.05). ^g^Significant differences between staphylococci-positive and -negative periodontitis patients.

### Frequency of staphylococci in the subgingival biofilm

**Figure 1:**
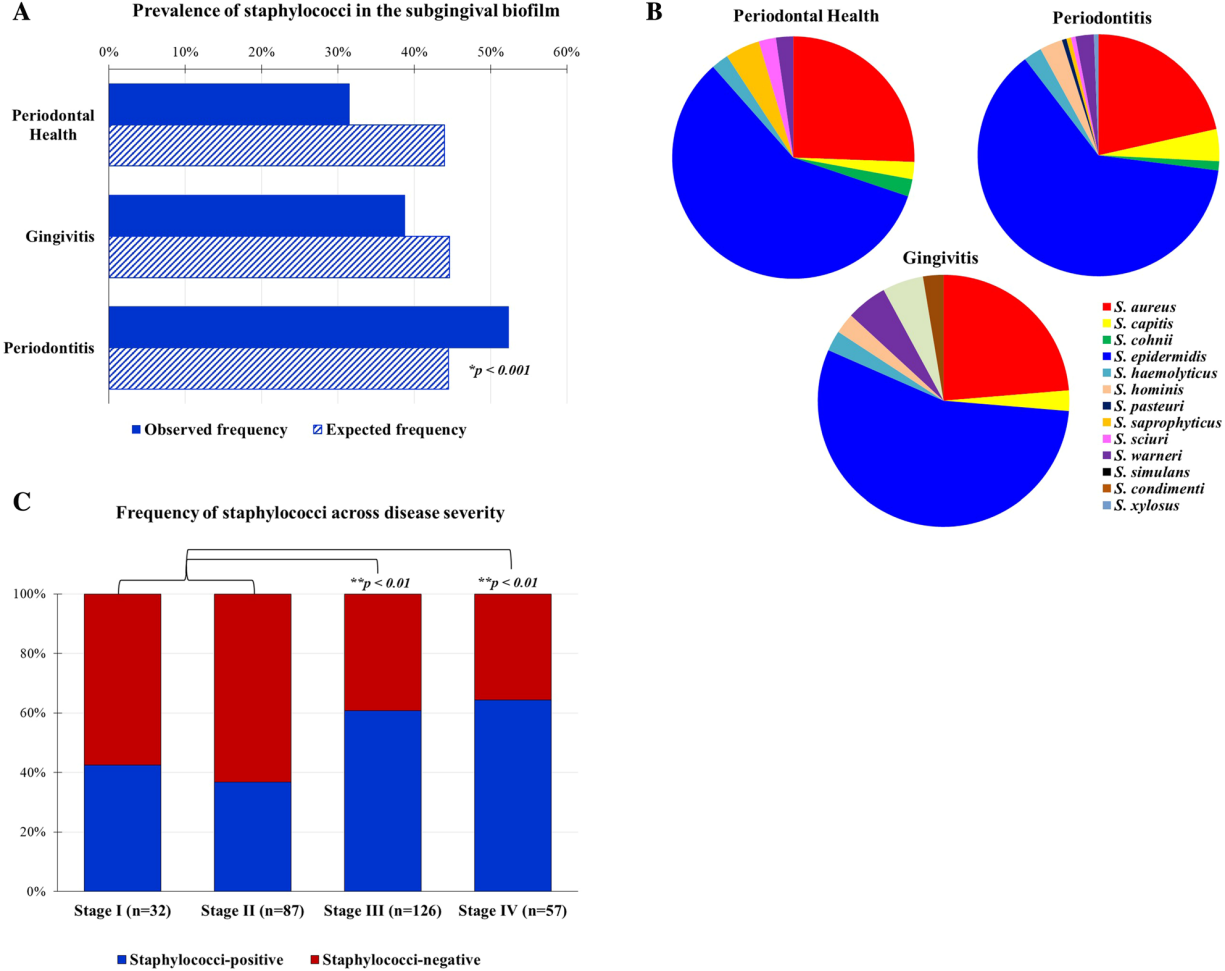
Prevalence of staphylococci species isolated from subgingival biofilm. (**A**) Observed and expected frequencies of staphylococci among individuals with distinct periodontal conditions. (**B**) Frequency of *Staphylococcus* species identified across clinical groups. (**C**) Increased prevalence of staphylococci in periodontitis patients with more severe disease^26,27^. Significant differences were observed by Chi-square and Fisher’s exact tests (*p < 0.001, **p < 0.01).

Two-hundred and fifty-one isolates obtained from 545 subgingival samples were identified as staphylococci. In 18 patients, two isolates of distinct staphylococci species were identified. Figure 1 shows the relative frequency distribution of *Staphylococcus* spp. across the clinical groups and disease severity. The overall observed frequency (52.4%) of staphylococci isolates was significantly greater than the expected frequency (44.5%) in the periodontitis group (Chi-square test, p < 0.001), but not in healthy or gingivitis patients (Fig. 1A). Regardless of the clinical condition, the predominant species were *S. epidermidis* (59%) and *S. aureus* (22%), followed by *S. capitis* (3.6%) and *S. warneri* (2.8%) at much lower prevalence. Nevertheless, the observed frequencies of *S. epidermidis* (69%), *S. aureus* (63%) and *S. capitis* (77%) were significantly higher than the expected frequency (33%) for patients with periodontitis (Chi-square test, p < 0.05). Of interest, a few isolates of *S. saprophyticus* predominated in subgingival samples from healthy individuals, whereas isolates of *S. condimenti, S. hominis, S. simulans, S. xylosus* were identified only in samples from periodontal diseases (Fig. 1B). Next, we examined the prevalence of staphylococci isolates among the 302 periodontitis patients according to disease severity^27^ (Fig. 1C). A significantly higher frequency of staphylococci isolates was observed in individuals with advanced forms of the disease (60.7% in stage III and 64.4% in stage IV) compared to mild periodontitis (42.4% in stage I and 36.7% in stage II). Given that significant differences among groups were observed for age, smoking, race and social-economic level, the detection of staphylococci isolates was also associated with these demographic parameters in all individuals and within each clinical group. Age was dichotomized into ≤ and > median age (36 years). No significant differences were seen in the distribution of staphylococci regarding these demographic features (Chi-square test, p > 0.05; data not shown).

### Antimicrobial susceptibility of oral staphylococci

**Figure 2:**
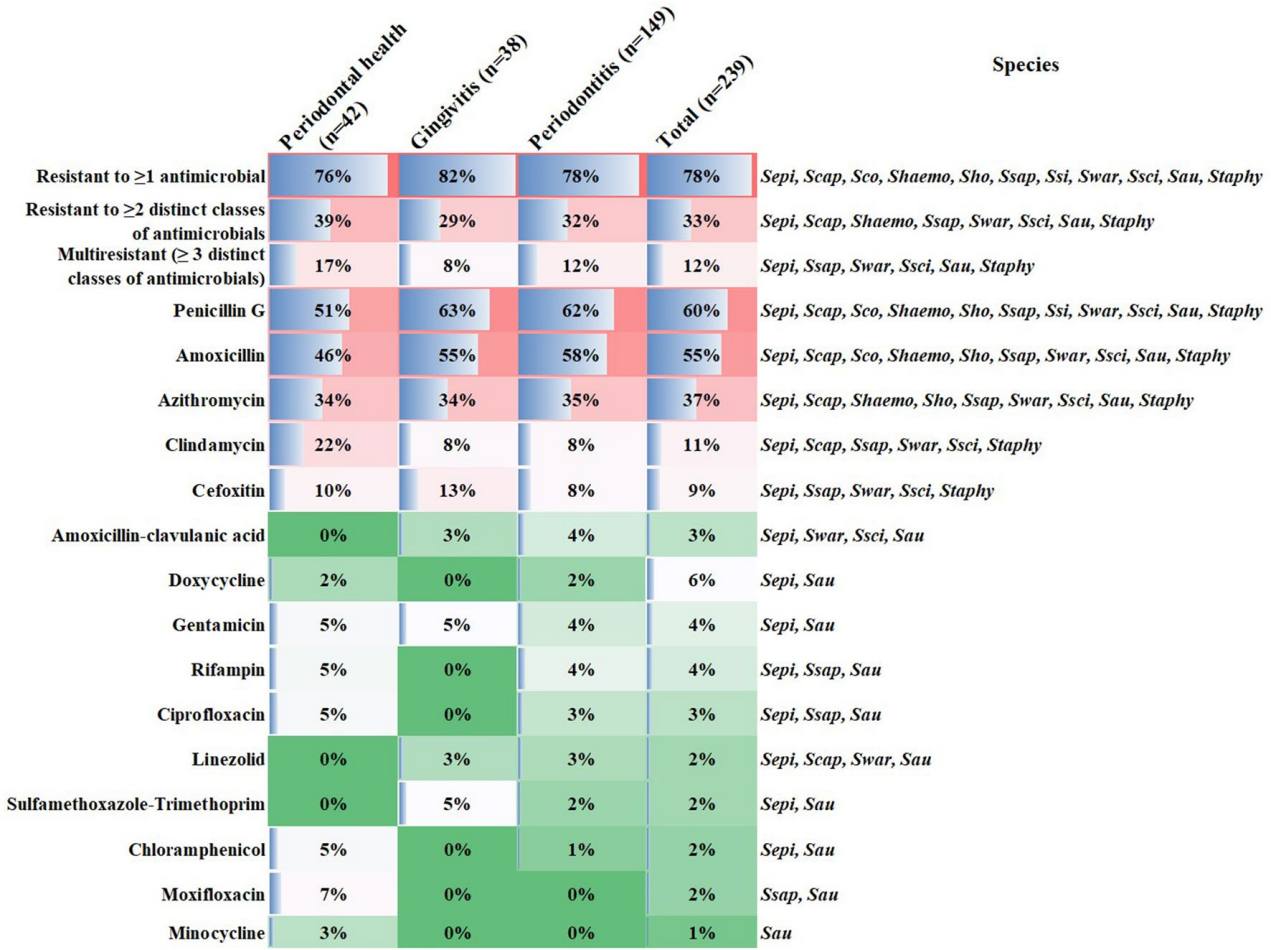
Antimicrobial susceptibility of oral staphylococci. Frequency distribution of staphylococci species with resistance and/or low susceptibility to antimicrobials across subgingival biofilm samples from individuals with periodontal health and diseases. *Sepi* (*S. epidermidis*), *Scap* (*S. capiti*),* Sco* (*S. cohnii*),* Shaemo* (*S. haemolyticus*),* Sho* (*S. hominis*),* Ssap* (*S. saprophyticus*),* Swar* (*S. warneri*),* Ssci* (*S. sciuri*),* Sau* (*S. aureus*),* Staphy* (*Staphylococcus* sp.).

Of the 251 strains isolated, 239 were recovered for the antibiogram testing. Most strains (78.2%) showed resistance and/low susceptibility to at least one antimicrobial, whereas only 12% were multidrug resistant, including the species *S. epidermidis, S. aureus, S. saprophyticus, S. warneri* and *S. sciuri*. Increased resistance rates were observed for penicillin G, amoxicillin and azithromycin (Fig. 2). Twenty-two methicillin-resistant staphylococci (MRS) were detected (9.2% cefoxitin-resistance), of which only five were *S. aureus* (MRSA). No significant differences were found for staphylococci susceptibility among strains from the three clinical groups or among stages of disease severity (Chi-square test, p > 0.05).

### Detection of *mecA* and virulence factor genes

Twenty-five staphylococci isolates carried the *mecA* gene (10.4%), including *S. epidermidis* (14), *S. aureus* (7), *S. capiti* (1), *S. cohnii* (1), *S. sciuri* (1), and a non-identified *Staphylococcus* sp. strain (1). Among *mecA* + strains, 72% were resistant to penicillin, 64% to amoxicillin and 44% were MRS, but only 24% were multidrug resistant and none were resistant to amoxicillin plus clavulanic acid (Table 2). In patients with periodontitis, > 70% of the *mecA* + strains were isolated from severe disease. Among MRS, 50% carried the *mecA* gene, while approximately 13% of strains resistant to penicillin or amoxicillin were *mecA*-positive. For the virulence factors tested, the genes encoding heat shock chaperonin (86.6%), the collagen-binding (30.3%) and elastin-binding proteins (25%) were the most detected (Table 2). All strains of *S. aureus* were *clfA*-positive. A significantly higher prevalence of *fnB* + isolates was observed in periodontitis patients in relation to healthy and gingivitis individuals (Chi-square test, p < 0.05). All virulence genes were detected in one strain of *S. epidermidis* and one strain of *S. aureus mecA* + isolated from patients with advanced periodontitis. High detection rates of *luxF/luxS-pvl* positive strains were associated with increased % of sites with deep pockets and attachment loss > 5 mm (Chi-square test, p = 0.026; data not shown). No relevant associations were seen between virulence factors and antimicrobial susceptibility, or between these characteristics and stages of disease severity.

**Table 2:** Distribution of *mecA* and virulence fator genes in subgingival staphylococci strains isolated from individuals with distinct periodontal conditions.

Genes	Periodontal health (n = 42)	Gingivitis (n = 38)	Periodontitis (n = 149)	Predominant species	Total (n = 239)
*mecA* +	3	4	18 (13^#^)	*Sep, Sau, Scap, Sco, Ssci, Staphy*	25
Resistant ≥ 2 antimicrobials	2	1	10*		13*
Resistant ≥ 3 antimicrobials	2	1	3		6*
Resistant to Penicillin	3	3	12		18
Resistant to Amoxicillin	3	3	10		16
Resistant to Cefoxitin	2*	3*	6*		11*
Virulence factors	(n = 37)	(n = 31)	(n = 141)		(n = 209)
* groEL*	33	22	119	All species	174*
* ebpS*	7	6	39	*Sep, Sau, Ssap, Swar, Scap, Sco, Ssci*	52
* cna*	8	12	43	*Sep, Sau, Scap, Ssap, Swar, Sco, Ssci, Sho*	63
* bbp*	0	0	4	*Sep, Sau*	4
* luxF/luxS-pvl*	0	2	10		12
* fnbA*	1	1	8		10
* fnbB*	1	2	25*	*Sep, Sau, Ssap, Swar, Sho*	28*

^#^Periodontitis Stages III/IV; *Significant differences among groups (Chi-square test, p < 0.05); *mecA* (low affinity penicillin-binding protein 2A); *ebpS* (elastin-binding protein), *cna* (collagen-binding protein), *fnbA* and *fnbB* (fibronectin-binding proteins A and B), *bbp* (bone sialoprotein-binding protein), *luxF/luxS-pvl* (Panton–Valentine leukocidin), *groEL* (chaperonin of the heat shock protein family).

## Discussion

Staphylococci are among the major opportunist pathogens responsible for global deaths and disability-adjusted life-years attributable to bacterial antimicrobial resistance^46^. Although the main sources of staphylococcal infections are skin and mucous membrane-related, the oral microbiota has been described as a potential reservoir of resistant staphylococci, particularly in immunocompromised, older and denture-wearing individuals, as well as patients with poor oral hygiene and oral infections^8^. Previously, our group reported a high mean prevalence of subgingival *S. aureus* (approximately 48%) by employing a culture-independent method^30^; however, viability, antimicrobial resistance and virulence could not be determined. In the current study, we used a culture approach to isolate, identify and partially characterize the resistance and virulence of subgingival staphylococci obtained from healthy and diseased periodontal conditions. Consistently with previous findings^12,13,17,19–22,30,47^, an overall prevalence of 45% of subgingival staphylococci was observed in this sample population, with a significantly increased frequency in severe periodontitis (60–64%). *S. epidermidis* and *S. aureus* were the predominant species detected. The frequency of staphylococci in the oral cavity may vary significantly among studies due to the distinct methods employed for their detection^8,10–12,19,47–49^. Investigators have reported a high prevalence of these microorganisms, specially *S. epidermidis* and *S. aureus*, in the dental plaque^13–15,17–22,31,50,51^; however, only a few found a significant association between these species and periodontitis^14,16,31^. The pathogenicity and MDR relevance of *S. aureus* in human infections has been extensively documented^52^. On the other hand, the less virulent and harmless CoNS, particularly *S. epidermidis*, have emerged as major pathogens of local and systemic bloodstream infections associated with indwelling medical devices^6^. The ability of biofilm formation and widespread antimicrobial resistance are key features of the CoNS regarding these infections^6,53^. In this study, low frequency rates of *S. capitis, S. warneri, S. hominis, S. simulans, S. condimenti* and *S. xylosus* were found in diseased individuals, corroborating previous studies^13,17,19,51,54–56^. The last two species are typically associated with fermented foods and rarely cause diseases in humans. In contrast, *S. epidermidis, S. capitis, S. warneri, S. hominis* and *S. simulans* are members of the clinically defined “*S. epidermidis* group”^53^. These species are main reservoirs of resistance genes, participating actively in the horizontal transmission of these genes to more virulent pathogens^6,53^, potentially including periodontal pathogens^57^. In addition, colonization of the dental biofilm by staphylococci may influence the virulence and structure of this complex oral community, triggering the change from a eubiotic biofilm to a dysbiotic one^32–34,58^. Using in vitro biofilm models, Lima et al.^33^ showed the ability of *S. aureus* to integrate and grow into a human-derived multispecies oral microbial community, affecting its composition. Furthermore, *S. aureus* co-aggregated specifically with *F. nucleatum* and *P. gingivalis*, but not other oral species. The *F. nucleatum-S. aureus* synergistic interaction involved specific fusobacterial adhesins and resulted in an increased expression of the gene regulator *sarA*, which is implicated in staphylococcal virulence. Similarly, Schnurr et al.^58^ demonstrated that different strains of *S. aureus* affected the growth of distinct oral species when integrated into a multispecies oral biofilm. Finally, the low frequency of commensal oral streptococci in the dental biofilm of elderly inpatients was strongly associated with increased rates of oral methicillin-resistant *S. aureus* (MRSA)^34^. Hence, the evident oral carriage of staphylococci, regardless of their direct impact on PDs, should not be underrated as a potential risk for cross-infections and antimicrobial resistance dissemination^7–9,59^.

In fact, high rates of resistance or reduced susceptibility to penicillin (60%), amoxicillin (55%) and azithromycin (37%) were found among subgingival isolates from all clinical groups. These antimicrobials are routinely prescribed in dental practices, which could lead to reduced clinical efficacy^60^. In contrast, most isolates were susceptible to cefoxitin and amoxicillin-clavulanic acid. Very similar antimicrobial susceptibility profiles of oral staphylococci^8,55,61–63^, including isolates from periodontitis patients^13,22,48,51^ have been reported. Fortunately, MDR was relatively low (12%) in our oral isolates compared to the high rates (> 20%) reported from other countries^49,55,61,64^. The overall detection of the *mecA* gene (10.5%) was consistent with the 9% rate of cefoxitin (methicillin) resistance. However, half of the MRS strains carried the *mecA* gene, while only 13% of the strains resistant to penicillin/amoxicillin were *mecA*-positive. Thus, increased resistance to penicillins, low carriage of *mecA* and high susceptibility to amoxicillin-clavulanic acid may suggest the predominance of other beta-lactam resistance mechanisms, such as the production of beta-lactamases encoded by *bla*Z genes, or the existence of other MR genes^57,59,65^. Carriage rates of *mecA* in oral staphylococci have been shown to range from 6 to 21% in most studies^8,14,22,49,55,63^, although no detection^48,50^ or very high frequencies are also reported in different populations^61,64,66^. Regardless of these variations or significant associations with periodontitis, these data support the oral cavity and the dental biofilm as potential reservoirs of beta-lactam resistance genes^57,59^.

The broad range of local and systemic diseases caused by staphylococci relates to their diverse armamentarium of virulence factors^2,6^ that promote adhesion to biomaterials and host extracellular matrices, invasion and damage of host tissue/cells, evasion of the immune system, inflammation and biofilm formation. In this study, virulence genes of the microbial surface component recognizing adhesive matrix molecules (MSCRAMMs) family, including *clfA, ebpS*, *can*, *fnb*, *bbp,* as well as the cytotoxin *luxF/luxS-pvl* and the heat shock chaperonin *groEL* were investigated. The clumping factor gene, which encodes a fibrinogen-binding surface protein with antiphagocytic properties, was detected in all strains of *S. aureus*. The *groEL* was the most prevalent virulence gene, followed by *ebpS* and *can*, whereas the *bbp* gene was detected only in strains from periodontitis. The bacterial metabolite groEL is a potent stimulator of inflammation, and it has recently been shown to impair osteogenic differentiation and promote the adipogenic capacity of periodontal ligament stem cells^67^. The *bbp* protein has been associated with osteomyelitis and arthritis^42^. This gene was reported to be predominant in specific MRSA lineages isolated from orthopedic infections in a Brazilian hospital^68^. In the current investigation, no significant differences in the detection of *ebpS, can, groEL* were found among clinical groups. Conversely, strains carrying the *fnB* and *luxF/luxS-pvl* genes were associated with periodontitis. Fibronectin-binding proteins are common in invasive staphylococci isolated from infective endocarditis and osteomyelitis^43,44,69^. On the other hand, staphylococci strains encoding the potent Panton-Valentine cytotoxin are generally much less prevalent^43,55,61^. PVL causes leukocyte destruction and it is correlated with dermonecrosis, furunculosis and community-acquired severe necrotic pneumonia^45^. Among the few studies on oral staphylococci, higher frequencies of MSCRAMM genes were reported in comparison to our data^22,64,69^, specifically in oral isolates of *S. aureus*. Furthermore, *pvl* was detected in more than 50% of staphylococci isolated from dental biofilm in Italians; however, no differences between actively progressing and non-actively progressing periodontal sites were observed^22^.

In conclusion, our data showed a high prevalence of penicillin-resistant staphylococci in the subgingival biofilm of individuals with periodontal health or disease. Strains carrying virulence genes related to tissue adhesion/invasion, inflammation and cytotoxicity indicate the pathogenic potential of these opportunists in the periodontal microenvironment. The role of distinct virulence and antimicrobial resistance phenotypes of oral staphylococci on periodontal health is still poorly understood^62^. Further studies focused on the relevance of these species on disease severity or progression and on response to antimicrobial periodontal therapy are warranted.

## Supplementary Information

Supplementary Table S1.

## Data Availability

The datasets generated and analyzed in this study are available from the corresponding author on a reasonable request.
