# Supplementary material for: Antimicrobial resistance and virulence of subgingival staphylococci isolated from periodontal health and diseases
Source: Sci Rep. 2023 Jul 18;13:11613. doi: 10.1038/s41598-023-38599-4 (PMC10354038; doi:10.1038/s41598-023-38599-4)
Supplement: Supplementary file 1 — Supplementary Table S1. [file 41598_2023_38599_MOESM1_ESM.docx]

**Antimicrobial resistance and virulence of subgingival staphylococci isolated from periodontal health and diseases**

**Supplementary information**

**Appendix Table S1**. Nucleotide sequences of staphylococci gene-specific oligonucleotide primers used in the study, PCR conditions and estimated amplicon sizes.

| **Target genes** | **Oligonucleotide primers (5´-3´)** | **Amplicon (bp)** |
| --- | --- | --- |
| Staphylococcal 16S rRNA | 5`-CCTATAAGACTGGGATAACTTCGGG-3`  5`-CTTTGAGTTTCAACCTTGCGGTCG-3` | 791 |
| *clfA* of *S. aureus*  (clumping factor A) | 5`-GCAAAATCCAGCACAACAGGAAACGA-3`  5`-CTTGATCTCCAGCCATAATTGGTGG-3` | 638 |
| *mecA* (low affinity penicillin-binding protein 2A) | 5`-TCCAGGAATGCAGAAAGACCAAAGC-3`  5`-GACACGATAGCCATCTTCATGTTGG-3` | 499 |
| Eubacterial 16S rRNA | 5`-AACTGGAGGAAGGTGGGGGAT-3`  5`-AGGAGGTGATCCAACCGCA-3` | 371 |
| *fnbA* (fibronectin-binding protein A) | 5`-GATTATTAACGCAGCAGTAG-3`  5`-GATAATTTAATGCCAGAGC-3` | 1362 |
| *fnbB* (fibronectin-binding protein B) | 5`-GTAACAGCTAATGGTCGAATTGATACT-3`  5`-CAAGTTCGATAGGAGTACTATGTTC-3` | 524 |
| *bbp* (bone sialoprotein-binding protein) | 5`-AACTACATCTAGTACTCAACAACAG-3`  5`-ATGTGCTTGAATAACACCATCATCT-3` | 575 |
| *ebpS* (elastin-binding protein) | 5`-CATCCAGAACCAATCGAAGAC-3`  5`-CTTAACAGTTACATCATCATGTTTATCTTT-3` | 186 |
| *cna* (collagen-binding protein) | 5`-GTCAAGCAGTTATTACACCAGAC-3  5`-AATCAGTAATTGCACTTTGTCCACT-3` | 423 |
| *luxF/luxS-pvl* (leukocidin Panton-Valentine toxin) | 5`-ATCATTAGGTAAAATGTCTGGACATGATCCA-3`  5`-GCATCAATGTATTGGATAGCAAAAGC-3` | 433 |
| *groEL* (heat shock chaperonin) | 5′-GAIIIIGCIG-GIGA(TC)GGIACIACIAC-3′  5′-(TC)(TG)I(TC)(TG)ITCICC(AG)AAICCIGGIGC(TC)TT-3′ | 550 |
